# Typologies of postnatal support and breastfeeding at two months in the UK

**DOI:** 10.1016/j.socscimed.2020.112791

**Published:** 2020-02

**Authors:** Emily H. Emmott, Abigail E. Page, Sarah Myers

**Affiliations:** a UCL Anthropology, University College London, 14 Taviton Street, London, WC1H 0BW, UK; b Department of Population Health, London School of Hygiene and Tropical Medicine, Keppel Street, London, WC1E 7HT, UK

**Keywords:** Breastfeeding, Mothers, Infant feeding, Social support, Latent class analysis, UK

## Abstract

There is extensive evidence to suggest that social support improves breastfeeding outcomes. Building on this evidence-base, public health services and interventions aiming to improve breastfeeding rates have primarily targeted informational and emotional support to mothers, reflecting an individual behaviour-change approach. However, mothers exist within a wider social network, and the characteristics of their broader support networks may be an important predictor of breastfeeding outcomes. Here we explore the typologies of postnatal support for mothers in the UK; a population with one of the lowest breastfeeding rates in Europe. Using retrospective data from an online survey (data collection period December 2017 - February 2018), we carry out a latent class regression (n = 432) to identify “clusters” of postnatal support in our data. Mothers in our sample were most likely to report receiving practical and emotional support from partners and maternal grandmothers, and breastfeeding information from health professionals. We identify three distinct typologies of postnatal support: 1) *Extensive* support, where mothers received support from a wide range of supporters including partners, maternal grandmothers, friends and health professionals, but mothers were the only ones to feed the infant; 2) *Family* support, where mothers received support from partners and maternal grandmothers, including with infant feeding, but less likely to receive support from health professionals; and 3) *Low* support, where mothers primarily received support from partners. 94% of women with *extensive* support were predicted to be breastfeeding at two months, followed by 48% of mothers in the *low* support group, and 13% in the *family* support group. Our findings highlight the complexities of family support and its potential impact on breastfeeding, as well as the significance of professional support. Overall, our results hint at the potential value for health professionals to engage with wider family in order to achieve *extensive* support for mothers.

## Highlights

We identify three types of postnatal support networks in our sample of UK mothers.
“Extensive support” mothers were supported by family, friends and professionals.
“Family support” mothers were mainly supported by family; less so by professionals.
“Low support” mothers reported lower support across all potential supporters.
Mothers who breastfed for > two months were most likely to report extensive support.

## Author contributions section

Emily H Emmott: Conceptualization; Data curation; Formal analysis; Investigation; Methodology; Project administration; Validation; Visualization; Roles/Writing - original draft; Writing - review & editing. Abigail E Page: Conceptualization; Data curation; Investigation; Project administration; Validation; Writing - review & editing. Sarah Myers: Conceptualization; Investigation; Project administration; Validation; Writing - review & editing.

## 1 Introduction

Breastfeeding has been associated with numerous physical health benefits for both mother and baby (Allen and Hector, 2005; Victora et al., 2016; Kramer and Kakuma, 2012), and the World Health Organisation currently recommends exclusive breastfeeding for 6 months (Victora et al., 2016; Kramer and Kakuma, 2012). However, despite continued efforts from the public health community (Bosi et al., 2016; Labbok, 2012), breastfeeding rates remain low in many developed countries (Bosi et al., 2016). In particular, breastfeeding rates in the UK are among the lowest in Europe (Bosi et al., 2016; UNICEF, 2013): A 2010 government survey estimated that 1% of UK women breastfeed exclusively at 6 months (Bosi et al., 2016; McAndrew et al., 2012), which is notably low compared to, for example, 18% in Netherlands and 28.5% in Spain (Bosi et al., 2016). In 2017/18, 42.7% of mothers in England were estimated to be providing breastmilk at 6–8 weeks (Public Health England, 2018a). In the aforementioned 2010 survey, around 80% of UK women who stopped breastfeeding within the first 6 weeks said they would have liked to breastfeed for longer (McAndrew et al., 2012), highlighting high levels of “unmet feeding goals.” Studies have repeatedly shown that such unmet feeding goals are often accompanied by a sense of guilt and failure (Burns et al., 2010), and problems associated with breastfeeding have been associated with postnatal depression (Brown et al., 2016).

Most mothers in the UK seem to share goals with the broader public health community in that they want to breastfeed, and breastfeed for longer. Why, then, are so many mothers currently struggling to meet their feeding goals? Studies suggest that the reasons for early breastfeeding cessation are multi-faceted and complex (Thulier and Mercer, 2009), spanning individual circumstance as well as socio-cultural factors. Many Western countries have a recent history of predominant formula feeding (Fomon, 2001), meaning the cultural norms for breastfeeding have been weakened. Many mothers are unable to draw on adequate breastfeeding support from family, friends, and sometimes from health professionals; and in some cases are discouraged by them from breastfeeding (Hoddinott and Pill, 1999; Fox et al., 2015; Taylor et al., 2019). Further, studies suggest mothers are often underprepared for the challenges of breastfeeding due to unrealistic messaging: Breastfeeding in the West is often promoted as being “natural,” which can lead to misconceptions that it is an instinctive/easy behaviour (Fox et al., 2015; Brown, 2016; Martucci and Barnhill, 2018; Williamson et al., 2012) – when in fact many women find breastfeeding challenging (Fox et al., 2015; Taylor et al., 2019; Brown, 2016; Williamson et al., 2012; Scott and Colin, 2002; Guyer et al., 2012). Overall, the lack of practical breastfeeding knowledge, combined with the shortage of breastfeeding support, leaves mothers vulnerable to breastfeeding challenges (Fox et al., 2015).

In response to such findings, public health services and interventions have focused on providing breastfeeding support, typically entailing provision of advice and information to mothers via health professionals or trained peer supporters (McFadden et al., 2017; Emmott and Mace, 2015). A recently updated Cochrane review of breastfeeding interventions found that support from health professionals and trained peers was associated with increased breastfeeding duration (McFadden et al., 2017). However, in the UK, the majority of randomised control trials (RCTs) have been ineffective at increasing breastfeeding initiation or duration (Hoddinott et al., 2011), and a systematic review of peer-support RCTs did not find evidence that such interventions improved breastfeeding outcomes (Jolly et al., 2012). While there are multiple causes leading to unsuccessful interventions (Hoddinott et al., 2011), we note that the current public health approach around breastfeeding typically focuses on a narrow pool of individuals as supporters, usually health professionals or trained peers, and sometimes fathers (Emmott and Mace, 2015). Interventions and policies in the UK are generally designed around individual behaviour change (Hoddinott et al., 2011), meaning they primarily target the mother. These approaches overlook the fact that mothers exist within a wider social network where friends and family influence maternal attitudes, knowledge, and experience of breastfeeding (Hoddinott and Pill, 1999; Fox et al., 2015; Emmott and Mace, 2015; Clifford and McIntyre, 2008; Schafer et al., 2016; Lavender et al., 2006). The core aim of our study is to broaden the focus of breastfeeding support to include wider family and friends, as well as health professionals, and examine its associations with breastfeeding outcomes in the UK.

### 1.1 The importance of wider support

Taking an evolutionary anthropological approach, we conceptualise breastfeeding as a “costly” maternal-investment behaviour (Emmott and Mace, 2015): Exclusive breastfeeding is estimated to require 450–700 kcals a day (Butte and King, 2005), increasing maternal nutritional requirements (Marlowe, 2003). Prolonged infant carrying, which often goes hand-in-hand with on-demand feeding, can be as energetically expensive as breastfeeding itself (Wall-Scheffler et al., 2007). On top of these energetic costs, breastfeeding often conflicts with other maternal activities leading to high opportunity costs (Emmott and Mace, 2015; Emmott et al., 2019; Quinlan and Quinlan, 2008; Hawkins et al., 2007; Cardenas and Major, 2005). For example, breastfeeding often conflicts with maternal labour: In developed populations, full-time maternal employment has been associated with a higher risk of breastfeeding cessation compared to part-time employment (Hawkins et al., 2007; Cardenas and Major, 2005; Johnston and Esposito, 2007; Fein et al., 2008). This conflict between breastfeeding and maternal labour is also observed cross-culturally, including in natural-fertility and subsistence populations (Emmott et al., 2019; Quinlan and Quinlan, 2008). In such populations, mothers are unable to simultaneously breastfeed and adequately provide for themselves and their infant on their own (Emmott et al., 2019; Quinlan and Quinlan, 2008). Consequently, mothers receive extensive support from a range of sources – including fathers, grandparents, aunts, uncles, and many non-relatives, leading to a communal childrearing system (Emmott et al., 2019; Quinlan and Quinlan, 2008; Du et al., 2019; Page et al., 2017).

While the childrearing systems in developed populations have arguably shifted from communal care to intensive parenting (Faircloth et al., 2014), we nonetheless hypothesise that mothers in the West require extensive support from a wide range of individuals for successful breastfeeding. This has, to some extent, been evidenced in qualitative studies of maternal experiences of breastfeeding support. For example, focusing specifically on the UK, in a study of 23 mothers and their families in North-West England, mothers discussed the lack of wider family support as a barrier to breastfeeding, while acknowledging the benefits of support from partners, family and friends (Lavender et al., 2006). Similarly, in a study of mothers attending Breastfeeding Cafés across England, mothers discussed how the views and actions of health professionals, friends and family all facilitated or undermined breastfeeding (Fox et al., 2015). These realities and experiences of mothers indicate that the availability of support from a broad range of individuals may be an important determinant of breastfeeding outcomes in developed populations.

However, at present, very little is known around the wider support networks of mothers in the UK: Available quantitative studies often take a dyadic approach to breastfeeding support, where a specific supporter, usually a health professional or a trained peer supporter, delivers breastfeeding information and advice to the mother (McFadden et al., 2017). While qualitative studies have identified a range of individuals as important sources of support, studies tend to focus on understanding the meanings and consequences behind instances of support, rather than build up a picture of the broader support system. While such studies are highly valuable, we are yet to develop a good understanding of the characteristics of maternal support networks in the UK, and how these associate with breastfeeding outcomes. From a public health perspective, for breastfeeding support initiatives to be maximally effective, they must complement and work with the existing support system around mothers. A crucial step is therefore to understand who supports mothers with infants, and whether there are any systematic patterns in the sources of support.

### 1.2 The importance of the types of support

An additional point to consider alongside who helps is *how* individuals help mothers. In social epidemiology, social support has been broadly conceptualised as a “resource transfer” from one person to another (Stansfeld et al., 2006). Such support has been categorised into *emotional* and *informational* support, where individuals are provided with information and encouragement which improves their skill, self-appraisal and self-esteem, and *practical* or *instrumental* support, where individuals are supported via direct or tangible actions (Stansfeld et al., 2006). While there is theoretical recognition that different types of support may lead to different health and behavioural outcomes (Stansfeld et al., 2006), studies rarely define or clarify the types of support being investigated. Social support is often described as complex and multifaceted (McFadden et al., 2017; Hoddinott et al., 2011), but the majority of studies focus on informational and/or emotional support, meaning our current understanding of practical support and breastfeeding outcomes is limited (Emmott and Mace, 2015). From an evolutionary anthropological perspective, we hypothesise that the different types of support – informational, emotional and practical – will have different pathways and effects on maternal breastfeeding (Tully and Ball, 2013):

1. *Informational support* involves knowledge transfer about infant feeding from the supporter to the mother. This may encourage breastfeeding via breastfeeding promotion, where discussion and knowledge of breastfeeding alters maternal perceptions of breastfeeding, normalising the activity (Emmott and Mace, 2015). Informational support may also increase maternal breastfeeding skill, thereby reducing the costs associated with breastfeeding. Note, the positive association between informational support and breastfeeding is contingent on the information being accurate, useful and helpful: Studies suggest conflicting and inaccurate information can undermine breastfeeding (Fox et al., 2015; Taylor et al., 2019). Further, mothers in England have reported receiving information which “pushed breastfeeding” (Burns et al., 2010). Such dictative informational transfers have been perceived as unhelpful and even harmful by mothers (Taylor et al., 2019; Burns et al., 2010), meaning information in itself is not necessarily supportive.
2. *Emotional support* is often expressed as empathy and connectedness between the supporter and the mother, which may or may not be related to infant feeding. Such support may signal the strength and availability of practical support mothers can draw on in future. Where mothers have a desire to breastfeed, knowing that “help is available” may promote breastfeeding by lowering the perceived opportunity costs of breastfeeding (Tully and Ball, 2013). Indeed, numerous studies on Western populations suggest emotional support is greatly valued by mothers, and seems to coexist with the perception of being able to rely on someone for support (Fox et al., 2015; Negron et al., 2013; Schmied et al., 2011). In contrast, the lack of emotional support may act as a barrier in accessing other forms of support. For example, in one qualitative study in England, lack of empathy from health professionals led to mothers being hesitant about asking for practical support (Fox et al., 2015).
3. *Practical support* involves direct action, for instance helping with childcare or providing financial assistance, which is likely to influence the costs and benefits of breastfeeding (Tully and Ball, 2013). Importantly, practical support is theorised to increase or decrease breastfeeding, depending on the local ecology and type of activity (Emmott and Mace, 2015; Emmott et al., 2019; Tully and Ball, 2013). Complementary support activities, such as helping with household labour, is thought to remove the need for mothers to carry out such activities, allowing mothers to invest more time and energy into breastfeeding. In contrast, conflicting support activities are those which clash with breastfeeding, thereby increasing the opportunity costs around breastfeeding. For example, high levels of infant care has been theorised and described to conflict with breastfeeding, which may create incentives for mothers to stop breastfeeding (Emmott and Mace, 2015; Lavender et al., 2006). In the UK Millennium Cohort Study, proxies of practical support from fathers and grandmothers, including paternal caregiving, have been associated with shorter breastfeeding duration (Emmott and Mace, 2015).

Overall, there is theoretical grounding and increasing indirect evidence to predict that the different types of support from different individuals may have different effects on breastfeeding. Consequently, examining the characteristics of the wider support system around the mother, rather than looking at individual support, could improve our understanding of how social support influences breastfeeding outcomes. For breastfeeding support initiatives to effectively address “unmet feeding goals” in the UK, a holistic understanding of the postnatal support around mothers could be crucial.

### 1.3 Current study

The aim of the current study is to improve understanding of the characteristics and consequences of the wider support around mothers in the UK. Specifically, using data from a convenience-sampled retrospective online survey, we explore the typologies of postnatal support and their associations with breastfeeding. We focus on support in the first few weeks after birth; a crucial period where mothers require high levels of support (Negron et al., 2013) which may be particularly important for breastfeeding outcomes (Hoddinott et al., 2011). We examine their associations with breastfeeding at two months (2 m), which is Public Health England's Key Performance Indicator for breastfeeding (Public Health England, 2018a). Importantly, we extend the typical view of key supporters to include family, friends and health professionals, and distinguish between the different types of support (informational, emotional and practical support).

## 2 Methods

### 2.1 Survey development and data collection

Here, we use data from a retrospective online survey. The survey was developed as part of a wider project on social support and maternal experience (https://osf.io/7kb5q/), hosted on Opinio (survey platform; http://objectplanet.com/opinio/). The final survey included questions on participant characteristics, household characteristics, child characteristics, birth experiences, support experiences and infant feeding experiences, taking 15–20 min to complete. An earlier version of the survey was independently tested and reviewed by two women unrelated to the project, who did not take part in the final survey. For more information on the survey, see https://osf.io/dbtpy/.

Women were eligible to take part in the survey if they currently lived and last gave birth in the UK, and their youngest child was under 24 months old at the time of the survey. As an exploratory study, we took an opportunistic approach and recruited participants through convenience-sampling between December 2017 and February 2018. While convenience-sampling is likely to introduce recruitment bias, it is cost and time efficient (Etikan et al., 2015). Survey adverts were posted on Twitter and Facebook (social networking sites), as well as Netmums (forum-based parenting website). Specifically for Facebook, survey adverts were posted on parenting-related Facebook groups with the permission of group administrators. The Facebook groups were diverse, including parenting groups, infant feeding support groups, and second-hand baby-item groups. Note, studies have shown that social media survey recruitment can lead to an increased proportion of middle-class participants (Topolovec-Vranic and Natarajan, 2016); however, this trend is not consistent, and it can be an effective way to recruit “hard-to-reach” populations (Topolovec-Vranic and Natarajan, 2016). We specifically targeted local Facebook groups based around the UK in an attempt to diversify our sample.

Survey adverts did not explicitly mention breastfeeding, but focused on support for new mothers and maternal experience (see supplementary information; SI). On the survey landing page, participants were informed that there would be some questions about infant feeding, with an explicit statement that it did not matter whether infants were breastfed or formula fed. While the survey could be accessed via mobile phone devices, participants were informed that it may be easier to take the survey on a personal computer/laptop. Multiple-entries were prevented using IP-address checks. Overall, 701 eligible mothers took part in the survey. Ethical approval for the survey was obtained from the UCL Research Ethics Committee (ref: 11479/001).

### 2.2 Analysis sample

For the current study, we explore postnatal support around mothers and its association with breastfeeding at 2 m. We therefore restrict our sample to those with breastfeeding information at 2 months (162 cases without breastfeeding information at 2 months removed from sample, including mothers whose infants were under 2 months old at time of survey). We further restrict our sample to mothers whose youngest child is a singleton (6 multiple birth cases removed) due to theoretical considerations, in that the support needs and pathways for multiple births may be different. This reduced our final eligible sample to n = 533. Data from our analyses for participants in our eligible sample who consented to publicly share their data are available online (N = 526; https://osf.io/7kb5q/).

### 2.3 Key variables used in the current study

#### 2.3.1 Postnatal support

Mothers were asked to retrospectively report whether they had received various types of support in the “first few weeks after birth.” For practical/instrumental support, we asked, *“Thinking back to the first few weeks after giving birth to your youngest child(ren), did the people listed below do any of the following things regardless of how helpful it was? Please tick all that apply.”* Specifically, we asked mothers if their partner, mother (maternal grandmother), father (maternal grandfather), brother(s), sister(s), partner's mother, partner's father and friends “did housework/chores around the house”, “fed my baby”, “generally looked after my baby.” Note, we asked mothers to report behaviours regardless of how helpful it was, in order to minimise reporting bias.

For informational support, we asked whether mothers received advice and/or information on “looking after my baby” and “breastfeeding”. In addition to the family and friends listed above, we asked whether mothers had received this information from several health-related professionals which included doctors (general practitioners), midwives, health visitors and breastfeeding mentors/peer supporters. In the UK, midwives are specialist antenatal-care nurses, engaging with mothers throughout pregnancy, during delivery, and soon after birth. Health visitors are specialist community public health nurses whose responsibilities include providing home visits, usually staring shortly before birth and continuing a few times until the child reaches age two – although there is variation in service provisions between geographic regions (Public Health England, 2018b). While breastfeeding mentors and peer support services are diverse, they are usually commissioned or voluntary supporters in the community who are specifically trained to provide breastfeeding information, advice, and support (The Breastfeeding Network, 2018).

Finally, for emotional support, we asked, “*Thinking back to the first few weeks after giving birth to your youngest children, overall, how emotionally* supported *did you feel by the following people?”* and asked how supported mothers felt by all the family members, friends, and health professionals mentioned above on a 5-point scale of “Very supported”, “Supported”, “Neither supported nor unsupported”, “Unsupported” and “Very unsupported.” For our analyses, “Very supported” and “Supported” were coded as receiving support, and “Neither Supported nor Unsupported”, “Unsupported”, and “Very unsupported” were coded as not receiving support.

For all support items, if participants selected “Not Applicable,” we interpreted this as an active indication that they did not receive that particular type of support, and was therefore coded as not receiving support. Non-response was treated as missing and removed from our analyses.

#### 2.3.2 Breastfeeding

Mothers were asked if they had ever breastfed the focal child (i.e. their youngest), and if so, how long for. This was used to derive two binary variables; *ever breastfed,* capturing breastfeeding initiation of the focal child, and *any breastfeeding at 2* *months*, indicating whether or not the mother was providing any breastmilk to the focal child at two months of age. 2 months was chosen as a cut-off point to reflect Public Health England's Key Performance Indicator, measuring breastfeeding in any form for 6–8 weeks (Public Health England, 2018a). Note, all mothers in our sample had initiated breastfeeding; likely a consequence of our convenience-sampling, discussed further in our limitations.

#### 2.3.3 *Socio-*demographics

In terms of family demographics, we present information on the mother's age at birth of the focal child, the focal child's sex, ethnicity, number of siblings, and mother's partnership status. As a measure of socio-economic position, we asked mothers about their highest qualification based on the UK education system. “Secondary Education” captures middle- and high-school qualifications including GCSEs, AS/A-Levels or equivalent (including O-Levels, Level 1–5 awards, Advanced Apprenticeships, International Baccalaureate, National Certificates and National Diplomas). “Higher Education” includes graduate degrees or equivalent (including graduate certificates and Level 6 awards). “Postgraduate Education” includes postgraduate degrees or equivalent (including PGCEs, Doctorates, Master's and Level 7–8 awards). If none of these applied, mothers could select “Other”, “Prefer not to say” or “No qualification.” As a measure of subjective socio-economic experience, mothers were also asked, “How would you describe your current financial situation?“. This was measured on a 5-point scale of “living comfortably”, “doing alright”, “just about getting by”, “finding it quite difficult”, and “finding it very difficult”.

### 2.4 Analysis

We first provide information of our sample characteristics, including descriptive statistics on the different types of postnatal support from different individuals reported by the mothers. We then present results from our latent class regression, an extension of latent class analyses, to explore the typologies of postnatal support and how this is associated with breastfeeding at 2 months in our sample of UK mothers. Latent class analyses can be used to identify how individuals cluster into discreet groups based on available data (Hagenaars and McCutcheon, 2002), and the patterns in the clustering can be used to infer the characteristics of the different classes (i.e., typologies).

#### 2.4.1 Latent class regression procedure

We began with a descriptive exploration of the data alongside exploratory latent class analyses to determine the support items and the number of classes to include in our final latent class regression model (see SI). Based on various model fit statistics and our subjective assessment of typology structures, our exploratory analyses led to a final model with three latent classes derived from practical, informational, and emotional support items from partners, mother's mothers (maternal grandmothers) and partner's mothers, as well as informational and emotional support items from friends, midwives and health visitors. Note, all support items from grandfathers, siblings, doctors, and breastfeeding supporters/mentors, as well as practical support items from friends, were removed from the final analyses as they did not improve model fit based on a range of indicators. A detailed outline of these exploratory analyses is provided in the SI.

Once the class numbers and key support items were identified, we conducted a “one-step” latent class regression model to explore how different typologies of postnatal support were associated with breastfeeding at 2 months. “One-step” latent class regression models are similar to running multinomial regressions with latent classes as the outcome, but the latent classes themselves are estimated simultaneously as the regression model (Bolck et al., 2004). In our preliminary analyses, we carried out a series of latent class regressions to select predictor variables for inclusion in our final model based on model fit (see SI).

Our final latent class regression included *breastfeeding at 2 months*, *focal child's sex*, and *number of focal child's siblings* as predictors, with the three latent classes as the outcome variable. All analyses were conducted in R v 3.5.2. All latent class analyses were performed using the R package *poLCA v 1.4.1* (Linzer and Lewis, 2011). In our exploratory analyses, some of our variable selection steps were performed using the R package *LCAvarsel v.1.1* (Fop and Murphy, 2017) (see SI).

## 3 Results

### 3.1 Sample characteristics

Table 1 outlines the sample characteristics of our full eligible sample (N = 533) and our final model sample with complete responses (N = 432 after listwise deletion). Most mothers in our sample had a partner at the time of the survey, who was the biological father of their child (full sample = 97%; final model sample = 99.1%). A high proportion of mothers reported post-graduate education (full sample = 43.2%; final model sample = 46.1%). In both samples, all mothers reported breastfeeding initiation, and a high proportion of mothers reported breastfeeding at 2 months (full sample = 82.9%, final model sample = 83.1%). This suggests there is an over-representation of breastfeeding mothers from higher socio-economic positions in our sample, likely due to our convenience-sampling method.

**Table 1:** Sample characteristics of our full eligible sample and final model sample.

Full analysis sample, N = 533	Mean	Range	SD	Final model sample, N = 432	Mean	Range	SD
Mother's age at birth of focal child	32.3	18–44	4.4	Mother's age at birth of focal child	32.5	18–44	4.25
Number of focal child's (older) siblings at time of survey	0.44	0–4	0.63	Number of focal child's (older) siblings at time of survey	0.44	0–4	0.62
	N	%			N	%	
Ever breastfed focal child (breastfeeding initiation)				Ever breastfed focal child (breastfeeding initiation)			
Yes	533	100		Yes	432	100	
No	0	0		No	0	0	
Any breastfeeding at 2 months				Any breastfeeding at 2 months			
Yes	442	82.9		Yes	359	83.1	
No	91	17.1		No	73	16.9	
Partner status^a^				Partner status			
Partnered with biological father of focal child	517	97.0		Partnered with biological father of focal child	428	99.1	
Partnered, not the biological father of focal child	4	0.8		Partnered, not the biological father of focal child	4	0.9	
Currently not partnered	12	2.3		Currently not partnered	0	0	
Focal child sex				Focal child sex			
Male	262	49.2		Male	217	49.8	
Female	271	50.8		Female	215	50.2	
Focal child ethnicity				Focal child ethnicity			
White	495	92.9		White	407	94.2	
Other	38	7.1		Other	25	5.8	
Mother's Highest Qualification				Mother's Highest Qualification			
Secondary Education	98	18.4		Secondary Education	72	16.7	
Higher Education	201	27.7		Higher Education	158	36.6	
Postgraduate Education	230	43.2		Postgraduate Education	199	46.1	
Other/Unknown	4	0.8		Other/Unknown	3	6.9	
Financial Situation				Financial Situation			
Living comfortably/doing alright	339	63.6		Living comfortably/doing alright	315	72.9	
Just about getting by	92	17.3		Just about getting by	84	19.4	
Finding it quite/very difficult	30	5.6		Finding it quite/very difficult	25	5.8	
Missing (inc. Prefer not to say)	72	13.5		Missing (inc. Prefer not to say)	8	1.9	
Maternal employment (at time of survey)^a^				Maternal employment (at time of survey)^a^			
Employed	427	73.7		Employed	371	85.9	
Not employed	79	13.6		Not employed	59	13.7	
Missing	73	12.6		Missing	2	0.5	

a These percentages do not add up to 100 due to rounding.

Table 2 outlines the proportion of mothers who reported receiving different types of support from different individuals, for our full sample and final model sample. In our data, partners and maternal grandmothers were most likely to be reported as providing practical and emotional support. For example, in our full eligible sample, 97.4% of fathers and 61.7% of maternal grandmothers were reported to have done housework/chores around the house, while 94.7% of fathers and 79.1% of maternal grandmothers were reported to have provided emotional support. Informational support was most likely to be reported from specialist nurses, with 81.5% of midwives and 72.1% of health visitors reported to have provided information on breastfeeding.

**Table 2:** Descriptive statistics of support items for our full eligible sample and final model sample. Percentages indicate the percentage of mothers who responded that they had received that type of support from the specified individual in the few weeks after birth.

	Full Analysis Sample (n = 533)						Final Model Sample (n = 432)					
	Practical Support			Informational Support		Emotional Support	Practical Support			Informational Support		Emotional Support
	Did housework/chores around the house	Generally looked after my baby (ies)	Fed my baby (ies)	Gave me advice/information about breastfeeding	Gave me advice/information about looking after my baby (ies)	–	Did housework/chores around the house	Generally looked after my baby (ies)	Fed my baby (ies)	Gave me advice/information about breastfeeding	Gave me advice/information about looking after my baby (ies)	–
Supporter	**%**	**%**	**%**	**%**	**%**	**%**	**%**	**%**	**%**	**%**	**%**	**%**
Partner	97.4	84.2	43.6	35.1	58.7	94.7	97.0	84.3	40.3	35.4	58.3	94.7
Mother's mother (maternal grandmother)	61.7	43.9	19.3	41.7	77.2	79.1	62.0	43.3	14.8	42.8	77.1	79.4
Mother's father (maternal grandfather)	22.0	15.9	5.6	1.6	23.4	53.2	24.6	17.6	4.6	1.9	23.4	53.8
Brother	3.3	3.0	2.3	2.5	5.9	25.1	3.5	3.3	1.6	2.6	5.9	25.4
Sister	12.4	13.4	5.9	17.3	25.1	42.4	12.8	14.6	5.6	18.3	25.5	
Partner's mother	30.7	23.5	10.4	24.9	50.0	54.6	29.9	22.2	7.6	23.8	49.1	53.7
Partner's father	8.7	10.5	4.8	1.9	11.4	33.8	8.9	10.3	3.3	1.4	10.3	33.3
Friend	11.3	7.8	4.5	52.5	63.8	77.2	11.1	8.1	3.5	53.5	64.1	74.1
GP/Doctor	–	–	–	18.8	29.9	38.7	–	–	–	19.0	29.2	38.4
Breastfeeding Mentor/Peer Supporter	–	–	–	44.1	17.7	37.8	–	–	–	44.6	17.0	38.1
Midwife	–	–	–	81.5	83.1	73.0	–	–	–	81.3	82.6	74.1
Health Visitor	–	–	–	72.1	81.4	65.9	–	–	–	73.6	81.7	66.2

### 3.2 Typologies of postnatal support

**Fig. 1:**
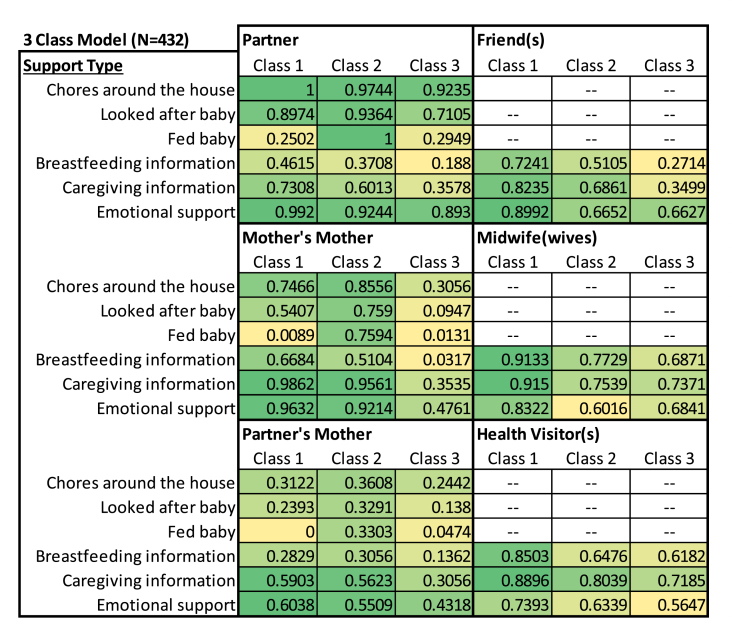
Predicted probability of support for each support item by class, from our final 3-class latent class regression (n = 432).

Here we present results from our final model; a three-class latent class regression including *any breastfeeding at 2 months*, *child's sex* and *number of siblings* as predictors of class membership. Fig. 1 shows the estimated probability of receiving support for each support type and source for each class (N = 432). Darker (green) colours indicate a relatively high probability of support, while lighter (yellow) colours indicate a relatively low probability of support. For example, mothers in Class 1 have a relatively low probability reporting infant feeding support from partners and grandmothers (P = 0.25 and P = 0.009, respectively), whereas mothers in Class 2 have a relatively high probability of reporting infant feeding support from partners and grandmothers (P = 1 and P = 0.76, respectively).

Based on this distribution of support across the classes, we constructed descriptions of the latent classes identified in our 3-class model (Table 3). We name Class 1 as the *extensive* support group, where mothers are likely to receive postnatal support from all individuals, but with low probability of help with infant feeding by the supporters. Class 2 is named the *family* support group, where mothers are likely to receive high support from partners and grandmothers, including help with infant feeding. However, mothers in this group are less likely to report receiving support from health professionals, particularly emotional support. Finally, Class 3 is named the *low* support group*,* where mothers are less likely to receive support across all the different supporters, particularly from their own mothers.

**Table 3:** Typologies of social support. Descriptions of classes identified in our final 3-class latent class regression model.

	Predicted class membership	Proposed typology name	Typology characteristics
Class 1	0.493	Extensive support group	Relatively high probability of postnatal support from all supporters, apart from practical support regarding infant feeding.
Class 2	0.178	Family support group	Relatively high probability of support from family members (partner and grandmothers), including practical support with infant feeding. Relatively lower probability of support from health professionals, particularly emotional support.
Class 3	0.329	Low support group	Relatively low support across all supporters, particularly maternal grandmothers, with most likely support being practical and emotional support from the partner.

### 3.3 Characteristics associated with the typologies of postnatal support

**Fig. 2:**
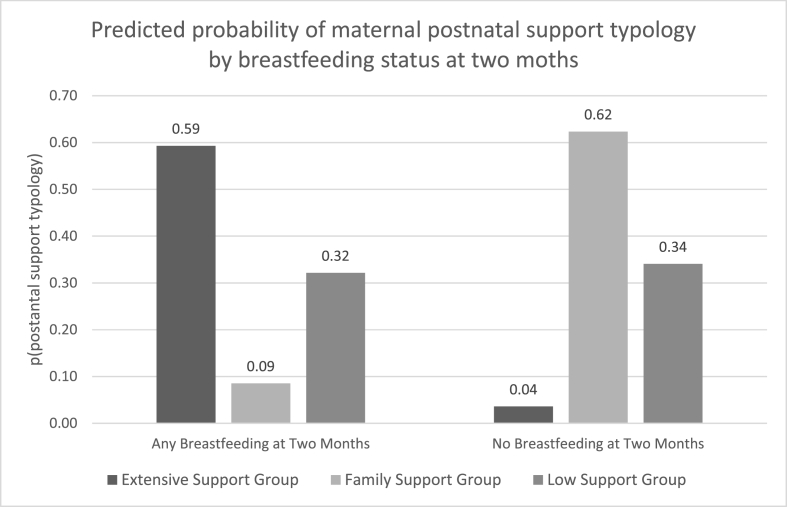
Predicted probability of postnatal support typologies by breastfeeding duration based on our final latent class regression model (n = 432), where the focal child is female and there are no other children (focal child's siblings) in the household.

Table 4 outlines how breastfeeding at 2 months, focal child's sex and number of focal child's siblings are associated with the typologies of postnatal support in our final model. Breastfeeding at 2 months was associated with a relative risk ratio (RRR) of 0.008 for being in the *family* support group compared to the *extensive* support group (95% CI = 0.001, 0.104; *p<*0.001), which means that women who breastfed for 2 months or longer were 99.2% less likely to be in the *family* support group compared to the *extensive* support group. Similarly, breastfeeding at 2 months was associated with a relative risk ratio of 0.057 for being in the *low* support group compared to the *extensive* support group (95% CI = 0.005, 0.616; *p* = 0.019), which means women who breastfed for 2 months or longer were 94.3% less likely to be in the *low* support group compared to the *extensive* support group. Fig. 2 displays the predicted probabilities of mothers' postnatal support typology by breastfeeding duration. Among mothers who breastfed for 2 months or longer, 59% were predicted to have *extensive* support, compared to 9% with *family* support, and 32% with *low* support. For mothers who breastfed for less than 2 months, 4% were predicted to have *extensive* support, compared to 62% with *family* support and 34% with *low* support. Overall, based on our model estimates, 94% of women with *extensive* support are predicted breastfeed for 2 months or longer, followed by 48% in the *low* support group and 13% in the *family* support group.

**Table 4:** Latent Class Regression results, similar in interpretation as a multinomial logistic regression. RRR = relative risk ratio.

N = 432	Family support group (Allen and Hector, 2005), compared to Extensive support group (0)				Low support group (Allen and Hector, 2005), compared to Extensive support group (0)			
	b	se	RRR	RRR 95% CI	b	se	RRR	RRR 95% CI
Intercept	2.853	1.232	17.3	1.404, 214.2	2.249	1.215	9.48	0.876, 102.5
Any breastfeeding at 2 months (ref. = no)	−4.791	1.288	0.008	0.001, 0.104	−2.861	1.213	0.057	0.005, 0.616
Child sex (ref. = female)	0.340	0.430	1.406	0.605, 3.264	−0.684	0.279	0.504	0.292, 0.871
Number of focal child's siblings	−0.397	0.370	0.673	0.325, 1.390	0.751	0.222	2.120	1.371, 3.276

Finally, our results also indicated that mothers were less likely to be in the *low* support group, compared to the *extensive* support group, if the focal child was male (RRR = 0.504; 95% CI = 0.292, 0.871; p = 0.015), and more likely to be in the *low* support group when there were more siblings in the household (RRR = 2.120; 95% CI = 1.371, 3.276; p = 0.001).

## 4 Discussion

Overall, mothers in our sample reported a wide range of postnatal support from different individuals. However, they were most likely to report practical and emotional support from fathers and maternal grandmothers, and informational support from health professionals. In our data, we find evidence of three distinct typologies of postnatal support: 1) mothers with *extensive* support were likely to report support from all supporters and across support types, bar infant feeding; 2) mothers with *family* support were likely to report support from family, including infant feeding, but were less likely to receive support from health professionals compared to mothers with *extensive* support; and 3) mothers with *low* support were less likely to report support across all supporters and support types. Mothers who breastfed for 2 m or longer were most likely to receive *extensive* support (59%), followed by *low* support (32%) and *family* support (9%)*.* It is important to note that these results are derived from a sample of mothers who all initiated breastfeeding, with a large proportion of mothers in higher socio-economic positions. Further, the causal directions between the typologies of postnatal support and breastfeeding for ≥2 m are unknown. Nonetheless, our findings demonstrate the potential for diversity in support experiences, even within a fairly homogenous group of UK mothers.

### 4.1 The importance of extensive support

Our results highlight the complex pathways between social support and breastfeeding: Compared to those with low support, receiving support from family may be associated with both an increased and decreased risk of early breastfeeding cessation – depending on whether family members provided practical infant feeding support. Mothers with *extensive* support reported very little infant feeding by others, while mothers with *family* support had a high probability of infant feeding by family. Such practical infant feeding support, often requiring introduction of the bottle, is likely to increase the probability of early breastfeeding cessation: Introduction of artificial nipples, bottle feeding of formula, as well as “exclusive” bottle feeding of breastmilk have all been associated with breastfeeding cessation (Fein et al., 2008; Jiang et al., 2015; Howard et al., 2003). Mechanistically, the introduction of the bottle/artificial nipples are associated with development of different sucking patterns in infants (Moral et al., 2010), and sucking patterns (both in infants and breast pumps) can influence milk production (Meier et al., 2011). While the causal direction between infant feeding by family and breastfeeding cessation is unclear due to the retrospective nature of our data, our findings reflect the importance of distinguishing the different types of support behaviours and how they relate to breastfeeding outcomes, adding to previous findings (Emmott and Mace, 2015; Cisco, 2017).

A further key difference between *extensive* and *family* support was the prevalence of informational and emotional support from health professionals – with a higher likelihood of professional support in the *extensive* support group associated with both lower help with infant feeding by family, and higher probability of breastfeeding at 2 m. Thus, our results also highlight the importance of professional support in the UK, where the weakened breastfeeding culture may mean mothers are particularly dependent on professionals for informational and emotional support. For example, for mothers who have access to family support, support from health professionals (thereby experiencing *extensive* support*)* may facilitate family members to support mothers without engaging with infant feeding, for instance by giving mothers the tools to direct family support to other activities. At the same time, lack of adequate professional support may mean mothers are not able to access or assess useful advice and information on how to breastfeed, feel confident about breastfeeding, and overcome breastfeeding challenges. To illustrate our point, in our open-text question at the end of our survey, one participant recounted her stressful experience of trying to seek professional support after experiencing challenges with breastfeeding – and, while she was supported by her family and friends, she was ultimately unable to continue after a month of pumping:

During pregnancy I assumed I would breastfeed and that it wouldn't be a problem. [Unfortunately] I found it difficult, my baby had problems latching and she kept losing weight after the first week before we were advised to combine with formula feeds. I sought help to improve latch from healthcare professionals but received little or no useful advice. In the worst cases I was given conflicting advice which may have contributed to the weight loss. With a lot of family and friend support I pumped for the first month and combined with formula to give my baby ‘the best’. … I felt unsupported and enormous guilt, I still do 5 months on!

It is important to note that the causal directions between these findings are unclear. For example, maternal postnatal support may change into a family-oriented system after early breastfeeding cessation (i.e., early breastfeeding cessation leading to the family support typology). Equally, practical infant feeding support from family may encourage breastfeeding cessation (i.e., family support typology leading to breastfeeding cessation). Nonetheless, these results reiterate why an individual behaviour-change approach to improving breastfeeding rates, exclusively targeting mothers, may not be particularly effective in the UK. In our sample of mothers, a range of family members and friends were providing different types of postnatal support. However, as family and friends are often not present when mothers receive professional breastfeeding support, many may lack the skills or knowledge to effectively support maternal breastfeeding goals (Hoddinott and Pill, 1999; Fox et al., 2015; Grassley and Eschiti, 2008). Previous studies have indicated that mothers require and value support from family and friends (Negron et al., 2013; Grassley and Eschiti, 2008), and that they are important determinants of breastfeeding outcomes (Clifford and McIntyre, 2008) – which, from an evolutionary perspective, influences the costs and benefits around breastfeeding behaviour (Emmott and Mace, 2015). In order to create *extensive* support for mothers, professional supporters may therefore need to engage with the wider support network around mothers. Our findings add evidence to support recent public health policy movements from targeting individuals to a family-centred approach, which are particularly gaining momentum in the UK regarding early-years universal support and interventions (House of Commons Health and Social Care Committee, 2019). However, the “family” in family-centred approaches are often limited to the nuclear family, namely mothers and their partners. Our results highlight the potential for looking “beyond the nuclear family”, in particular by including maternal grandmothers who were the largest providers of practical and emotional support after partners.

### 4.2 Low support and breastfeeding

In our final model, we also found that mothers in the *low* support group were less likely to breastfeed for 2 months or longer compared to the *extensive* support group, but more likely to breastfeed compared to the *family* support group. Given that social support is a known determinant of breastfeeding, it is puzzling to find that the probability of early breastfeeding cessation is *lower* for mothers receiving low support compared to mothers receiving family support. One possibility is that some mothers with *low* support did not “require” support to maintain breastfeeding. To exemplify this, one participant told us:

My husband has supported me greatly and the reason our parents and friends have not supported us massively is because luckily everything has been very straightforward for us and I have not really needed much support. Having a child is relentless and draining and so having my friends listen to me moan about being tired is about all the support I have needed.

This could also explain why higher parity (i.e., high number of focal child's siblings) predicted *low* support in our final model: Some mothers with previous experiences of infant feeding may have had more knowledge and skill to maintain breastfeeding without having to draw on much support from family, friends and health professionals. Several participants told us how their experiences from their first child meant they required less support, including one participant who wrote:

I feel as this was my second child I had a greater confidence and less support was needed - had I been answering after the birth of my first child the answers may have been very different …

However, given that *low* support is associated with a higher probability of early breastfeeding cessation compared to *extensive* support, we hypothesise that our *low* support group is a heterogeneous typology which also includes women who required but did not receive adequate support from their social network and professionals. Indeed, some of our participants recounted their often heart-breaking experiences of not receiving the support they needed. For example:

My experience of postnatal care and infant feeding support were so completely negative I am still struggling to process it. It was not just negative, but bullying to the point of abusive. At my most vulnerable, I felt pressurised, bullied, manipulated and humiliated by institutions I trusted, such as the NHS and the NCT. I was made to feel that my mental health was not only unimportant but in fact necessarily expendable for the sake of my baby. My own self-destructive and self-punishing instincts were enabled to the point of my baby starving and my developing serious postnatal depression. I was convinced I was the world's worst mother by the time my daughter was only a few weeks old.

Our results also indicated that mothers with female infants were more likely to be in the low support group compared to mothers with male infants. The sex ratio of children in our sample was relatively even at 101 (49.8% girls, 50.2% boys), meaning this result is not an artefact of a sex-ratio skew. While the mechanisms around this finding is unclear, both biological mechanisms and social norms in the UK may encourage allomothers to provide more support to mothers with male infants: Biologically, mothers with male infants produce more breastmilk, and produce breastmilk with greater nutritional content, compared to mothers with female infants (Powe et al., 2010). Giving birth to boys is also associated with increased risk of obstetric complications (Brettell et al., 2008), pre-eclampsia (Elsmén et al., 2006), gestational diabetes (Di Renzo et al., 2007), and postnatal depression (Myers and Johns, 2019). Overall, the greater “biological costs” associated with boys could mean mothers with male infants require and receive more social support than mothers with female infants. Further, studies have suggested son-biases in fathering in the UK (Emmott and Mace, 2018) which may reflect son-biases in wider caregiving, although female-biases in grandparental support have also been reported (Tan et al., 2010). Given the uncertainties around possible mechanisms, we caution against drawing strong conclusions regarding the association between infant sex and the typology of social support.

Overall, we hypothesise two different pathways to *low* support: some women may experience low support as they do not require the support, while others may experience low support as they are unable to access support. This may explain why, as a typology of postnatal support, *low* support is associated with a lower probability of breastfeeding for 2 months or longer compared to *extensive* support, but a higher probability breastfeeding for 2 months or longer compared to *family* support. In our current study, we are unable to explicitly test this hypothesis *post-hoc*, as we do not have the necessary information on the differential need for support. In future, it may be useful for researchers to attempt to distinguish between low support by unmet support needs.

## 5 Limitations

First, our data are the product of convenience-sampling, and mothers in our sample were more likely to be partnered, be from a White ethnic background, and have a higher education level than the general UK population. Importantly, all mothers in our final sample had initiated breastfeeding. While we hope our findings are useful in informing research with mothers from more diverse backgrounds, our current findings should not be directly extrapolated to different groups of mothers in the UK and beyond. In future, we recommend researchers carry out purposive sampling, including targeting women who did not initiate breastfeeding, or targeting women from different ethnic backgrounds.

Second, the sources of support and the types of support in our study was pre-defined, meaning we may have overlooked key supporters and support behaviours. While our survey questions were theoretically informed, the typologies we have identified are limited by the supporters and support items included in our survey. To address this issue, future studies may benefit from social network mapping and analysis, with more observational and ethnographic studies clarifying what types of support mothers actually receive in the postnatal period.

Finally, our data was collected retrospectively, meaning there is a risk that self-reported support may be coloured by breastfeeding experience. Further, we lack granular information on the frequency/timings of postnatal support, and for mothers who stopped breastfeeding before 2 months, it is unclear whether support came before or after breastfeeding cessation. Consequently, the causal pathways between the associations we find in this study are unclear. For instance, while receiving help with infant feeding may induce breastfeeding cessation, breastfeeding cessation may also create opportunities for supporters to provide help with infant feeding. Nonetheless, our study serves as the first step in understanding the broader postnatal support networks around mothers, and we hope researchers with opportunities for prospective data collection will build on our findings.

## 6 Conclusions

Our results add to a growing body of literature highlighting the importance of family, friends and health professionals in breastfeeding outcomes (Clifford and McIntyre, 2008). Based on evolutionary anthropological theory, we predicted that long-term breastfeeding is dependent on extensive support from multiple individuals (Quinlan and Quinlan, 2008). In support, we found that mothers with extensive postnatal support from family, friends and health professionals were most likely to breastfeed for 2 months or longer, compared to those with low support or family-biased support. As such, our findings reflect the importance of a family-centred approach to support breastfeeding in the UK, and the potential in looking “beyond the nuclear family.” Our results also indicate the need for the public health literature to take a more nuanced approach to social support, including consideration of the different forms of support and the differential impact they may have.
