# Supplementary material for: Typologies of postnatal support and breastfeeding at two months in the UK
Source: Soc Sci Med. 2020 Feb;246:112791. doi: 10.1016/j.socscimed.2020.112791 (PMC7014584; doi:10.1016/j.socscimed.2020.112791)
Supplement: Multimedia component 1 [file mmc1.docx]

Supplementary Information

Contents

[Further Information on Data Collection 1](#_Toc23527079)

[1. Survey Advert Examples 1](#_Toc23527080)

[Further Information on Data Analysis 2](#_Toc23527081)

[2. Initial selection of support items 2](#_Toc23527082)

[3. Initial selection of number of classes 3](#_Toc23527083)

[4. Further selection of support items 4](#_Toc23527084)

[5. Final selection of class numbers for LCA 5](#_Toc23527085)

[6. Selecting variables for latent class regression analyses 8](#_Toc23527086)

[References 9](#_Toc23527087)

# Further Information on Data Collection

## Survey Advert Examples

Below are examples of survey adverts and recruitment messages which were sent/posted. Note, the full survey is available elsewhere (<https://osf.io/dbtpy/>).

**Messages for facebook group admins:**

Dear X,

We are researchers investigating the support available to mothers with young infants, how they experience this support, and the impact this has on maternal experience. We are seeking mothers who have given birth less than 24 months ago in the UK and are willing to complete a short online survey (https://opinio.ucl.ac.uk/s?s=51442 ). We hope our findings will contribute to public health understandings of how best to support mothers during the first few years of their children’s lives. We were wondering if you would be willing to share the link to our study on your website/Facebook page?

The research has received ethical approval from the UCL Research Ethics Committee (Project ID Number): 11479/001. All participants will remain anonymous and data will be securely stored in line with current EU guidelines. More information regarding the study can be found on the first page of the online survey (https://opinio.ucl.ac.uk/s?s=51442 ).

Many thanks,

**Twitter/facebook post:**

Are you a mother of a 0-24m old, and gave birth in the UK? Take our survey to help understand how to support new mothers ☺ (https://opinio.ucl.ac.uk/s?s=51442) UCL Ethics ID 11479/001

We're looking for mothers of a 0-24m old kids, who gave birth in the UK. The survey will help understand how to support new mothers https://opinio.ucl.ac.uk/s?s=51442 UCL Ethics ID 11479/001

# Further Information on Data Analyses

Our aim for the preliminary analyses was to reduce the number of support items in the model to avoid over-fitting, as well as select the most meaningful number of latent classes. Outlined below is the exploratory method which led to our final model presented in the main manuscript.

## Initial selection of support items

In order to reduce the number of variables in the final models, as an initial step, we first conducted exploratory analyses to examine (1) how each social support type (practical, informational, and emotional support) clusters in our data, and (2) which supporters best predict these clusters.

For each support type, we first ran a series of latent class analyses using *poLCA v.1.4.1* (Linzer and Lewis 2011) with all available variables. We compared BIC model-fit values between 2, 3, 4, 5 and 6 class models to establish the appropriate number of latent classes within each support type. Using this estimated number of latent classes, we then conduced variable selection using *LCAvarsel v.1.1*, with the swap-stepwise backward/forward selection method (Fop and Murphy 2017). All analyses were carried out in *R v.3.5.2* (Team 2018).

This provided a list of supporters that best predicted the latent classes within each support type, indicating these supporters are relatively “important” to keep in our final series of analyses (see Table S2). Based on these results, we removed maternal/paternal grandfather and GPs from the next stage of our analyses.

Table S2: Results summary from initial latent class variable selection for practical support, informational support and emotional support.

| Support Type | **Practical Support** | **Informational Support** | **Emotional Support** |
| --- | --- | --- | --- |
| **N** | 487 | 470 | 449 |
| **Number of classes in best-fit model** | 4 | 5 | 3 |
| **Supporters included after model selection** | Mother’s mother (feeding)  Partner’s mother (care)  Sister (chores)  Friend (feeding, care, chores) | Partner (breastfeeding)  Mother’s mother (breastfeeding)  Sister (breastfeeding)  Midwife (breastfeeding)  Health Visitor (breastfeeding)  Peer supporter/ mentor (breastfeeding, caregiving) | Mother’s mother  Partner’s mother  Brother  Sister  Friend |

##

## Initial selection of number of classes

To decide on the appropriate number of classes for our final latent class analysis (LCA) model, we ran a series of LCA models on practical, informational and emotional support items from partners, mother’s mother (maternal grandmothers), partner’s mother, brothers, sisters, friends, midwives, health visitors and peer supporters/mentors (N(complete cases)=418). We compared BIC, AIC, aBIC and AICc values for 1, 2, 3, 4, 5 and 6 class models. While AIC and aBIC suggested that a 6-class model may be the most appropriate number of latent classes, entropy was at 0.7 meaning class boundaries maybe somewhat “fuzzy” and less distinct (Celeux and Soromenho 1996) (see Table S3). The BIC value suggested that a 5-class model may be most appropriate, while AICc and Entropy values suggested a 4-class model may be preferable. Note, AICc has been recommended for model selection with relatively small sample sizes (Burnham and Anderson 2002).

Table 3: Model fit values for latent class models with 1 to 6 classes in our initial model. "Best fit" values are in bold.

| **Number of classes (N=418)** | **Log likelihood** | **df** | **BIC** | **AIC** | **aBIC** | **AICc** | **Likelihood ratio** | **Entropy** |
| --- | --- | --- | --- | --- | --- | --- | --- | --- |
| **1** | -11250.9 | 534 | 22788 | 22591.74 | 22645.15 | 22833 | 13001.65 | - |
| **2** | -10672.9 | 488 | 21924.59 | 21527.71 | 21635.7 | 22015.59 | 12059.08 | 0.755 |
| **3** | -10360.3 | 442 | 21592.05 | 20994.55 | 21157.13 | 21729.05 | 11579.64 | 0.816 |
| **4** | -10123.6 | 396 | 21411.26 | 20613.14 | 20830.3 | **21594.26** | 11206.18 | **0.846** |
| **5** | -9969.98 | 350 | **21396.69** | 20397.95 | 20669.71 | 21625.69 | 10972.79 | 0.79 |
| **6** | -9861.22 | 304 | 21471.81 | **20272.45** | **20598.79** | 21746.81 | **10809.49** | 0.702 |

## Further selection of support items

A visual inspection of the estimated probability of receiving support by class in the 4-class model suggested that (see Figure SI1):

1. Support from siblings (i.e., brothers and sisters) did not notably vary between classes, apart from a few support items – therefore, brothers and sisters could be removed from the analyses
2. Practical support levels were generally low for brothers, sisters and friends across classes, with little variation – therefore, practical support items from brothers, sisters and friends could be removed from the analyses
3. Overall, class 1 and class 3 is very similar with minimal differences. We suspected that our results may be influenced by the fact that a substantial number of mothers do not have access to brothers/sisters/peer supporters, thereby an extra class may be emerging between mothers who have access to brothers/sisters/peer supporters. With this, brothers, sisters, and peer supporters could be removed from the analyses, and while model fit is better for a 4-class model (as it explains more variance), a 3-class model may be more meaningful.

Figure S1 Estimated probability of receiving support for each type/supporter by class, for the 4-class model. (N=418)

To examine whether there is a case to remove further support items, we ran the following models and compared model fit indices (N=418):

- Model 1: Full model including practical, informational and emotional support from partners, grandmothers, siblings, friends and health professionals
- Model 2: Removal of all sibling support items (1)
- Model 3: Removal of practical support from siblings and friends from the full model (2)
- Model 4: Removal of peer support from the full model (3)
- Model 5: Removal of peer support and siblings from the full model (1 & 3)
- Model 6: Removal of practical support from friends, and peer support and siblings from the full model (1, 2 & 3)

Model fit indicators suggested that the 4-class model without siblings and peer supporters, and without practical support items from friends, was a better fit (Table S4). The same patterns were replicated in the 3, 5 and 6 class models (results not shown). Therefore, the final LCA models included practical, informational and emotional support items from partners, maternal grandmothers and paternal grandmothers, as well as informational and emotional support items from friends, midwives and health visitors.

Table S4 Model fit indicators for 4-class model with various support items removed (N=418). Model 1: Full model including practical, informational and emotional support from partners, grandmothers, siblings, friends and health professionals. Model 2: Removal of all sibling support items. Model 3: Removal of practical support from siblings and friends from the full model. Model 4: Removal of peer support from the full model. Model 5: Removal of peer support and siblings from the full model. Model 6: Removal of practical support from friends, and peer support and siblings from the full model.

| **4 Class Model** | **Log likelihood** | **df** | **BIC** | **AIC** | **aBIC** | **AICc** | **Likelihood ratio** | **Entropy** |
| --- | --- | --- | --- | --- | --- | --- | --- | --- |
| **Model 1** | -8104.2 | 235 | 17312.89 | 16574.4 | 16732.18 | 17495.89 | 11165.51 | 0.89 |
| **Model 2** | -6574.53 | 283 | 13963.86 | 13419.07 | 13535.46 | 14098.86 | 8106.177 | 0.878 |
| **Model 3** | -7286 | 271 | 15459.21 | 14865.99 | 14992.74 | 15606.21 | 9529.104 | **0.904** |
| **Model 4** | -7387.87 | 247 | 15807.81 | 15117.74 | 15265.18 | 15978.81 | 9732.852 | 0.886 |
| **Model 5** | -5862.83 | 295 | 12468.02 | 11971.66 | 12077.71 | 12591.02 | 6691.083 | 0.842 |
| **Model 6** | -5554.08 | 307 | **11778.11** | **11330.17** | **11425.87** | **11889.11** | **6080.187** | 0.882 |

## Final selection of class numbers for LCA

With the support items for the final model settled, we re-examined the appropriate number of classes in our LCA models (N=466). As before, while some of the model fit indicators suggest the 5-class and 6-class models are a better fit, the entropy values are notably below 0.8 which suggests the classes are not clearly defined and they are not suitable for our final model.

Table 5: Model fit values for latent class models with 1 to 6 classes after final removal of support items. "Best fit" values are in bold.

| **Number of classes (N=466)** | **Log likelihood** | **df** | **BIC** | **AIC** | **aBIC** | **AICc** | **Likelihood ratio** | **Entropy** |
| --- | --- | --- | --- | --- | --- | --- | --- | --- |
| **1** | -7746.9 | 552 | 15665.55 | 15547.8 | 15579.84 | 15692.55 | 8185.482 | -- |
| **2** | -7288.82 | 524 | 14927.51 | 14687.64 | 14752.91 | 14982.51 | 7357.397 | 0.748 |
| **3** | -7063.08 | 496 | 14654.14 | 14292.15 | 14390.65 | 14737.14 | 6957.519 | **0.795** |
| **4** | -6916.92 | 468 | 14539.94 | 14055.84 | 14187.56 | **14650.94** | 6675.97 | 0.787 |
| **5** | -6821.88 | 440 | **14527.98** | 13921.76 | 14086.71 | 14666.98 | 6499.073 | 0.593 |
| **6** | -6734.53 | 412 | 14531.4 | **13803.06** | **14001.24** | 14698.4 | **6336.309** | 0.552 |

We further inspected the 3-class model and the 4-class model, to assess which class number would be most appropriate for our final analyses. We noted that one of the classes in the 4-class model had a small proportion of mothers attributed to that class (4-class model estimated class population shares; Class 1: 0.092, Class 2: 0.4456, Class 3: 0.2559, Class 4: 0.2065). Given our relatively small sample size and low frequency of mothers not breastfeeding at 2m, such a small class may be problematic for further analyses. A visual inspection of 3-class and 4-class models (Figure S2 and Figure S3) suggested that the classes between models are relatively consistent, with Class 1 and Class 4 in the 4-class model merging in the 3-class model (3-class model estimated class population shares; Class 1: 0.2057, Class 2: 0.4771, Class 3: 0.3172). Therefore, we decided to proceed with a 3-class model for our final analyses.

Figure S2: Estimated probability of support item response by class, for a 4-class model using our final support items.

Figure S3: Estimated probability of support item response by class, for a 3-class model using our final support items.

## Selecting variables for latent class regression analyses

To select predictor variables for our latent class regression analysis, we added each variable to the 3-class model without any predictors (i.e., null model; N=424). Compared to the null model, addition of financial situation, child’s ethnicity, and maternal employment did not improve model fit across any model-fit indicators (Table S6). Therefore, these variables were removed from our final latent class regression analyses. Further, models with mother’s qualification and mother’s age at birth experienced estimation issues without substantially improving model fit, and were therefore removed from the final models to reduce risk of over-fitting.

Table S6: Model fit values for our final latent class model (3-class; Null) and a series of latent class regressions with an addition of a independent predictor to the null model. "Best fit" values are in bold.

| **Model (N=424)** | **Log likelihood** | **df** | **BIC** | **AIC** | **aBIC** | **AICc** |
| --- | --- | --- | --- | --- | --- | --- |
| **Null model (4 class model)** | -5694.71 | 341 | 11891.54 | 11555.41 | 11628.15 | 11974.54 |
| **+ Breastfeeding at 2m** | -5673.1 | 339 | **11860.43** | **11516.2** | **11590.69** | **11945.43** |
| **+ Mother’s Highest Qualification** | -5688.12 | 335 | 11914.66 | **11554.24** | 11632.24 | 12003.66 |
| **+ Financial Situation** | -5689.78 | 335 | 11917.98 | 11557.55 | 11635.55 | 12006.98 |
| **+ Child Gender** | -5688.17 | 339 | **11890.57** | **11546.35** | **11620.84** | 11975.57 |
| **+ Child Ethnicity** | -5693.03 | 339 | 11900.28 | 11556.05 | 11630.55 | 11985.28 |
| **+ Mother’s age at birth** | -5691.93 | 339 | 11898.09 | **11553.86** | 11628.35 | 11983.09 |
| **+ Maternal Employment (at time of survey)** | -5692.73 | 337 | 11911.78 | 11559.46 | 11635.7 | 11998.78 |
| **+ Number of focal child’s siblings** | -5690.62 | 339 | 11895.46 | **11551.23** | **11625.73** | 11980.46 |

## References

Burnham, K P, and D R Anderson. 2002. *Model Selection and Multimodel Inference. A Practival Information-Theoretic Approach. Second Edition.* (Springer-Verlag: New York).

Celeux, Gilles, and Gilda Soromenho. 1996. 'An entropy criterion for assessing the number of clusters in a mixture model', *Journal of classification*, 13: 195-212.

Fop, M. , and T. B. Murphy. 2017. 'LCAvarsel: Variable selection for latent class analysis. R package version 1.1.'.

Linzer, Drew A., and Jeffrey B. Lewis. 2011. 'poLCA: An R Package for Polytomous Variable Latent Class Analysis', *2011*, 42: 29.

Team, R Core. 2018. 'R: A language and environment for statistical computing.', *R Foundation for Statistical Computing, Vienna, Austria. URL* [*https://www.R-project.org/*](https://www.R-project.org/).
